# Feeding behavior and activity levels are associated with recovery status in dairy calves treated with antimicrobials for Bovine Respiratory Disease

**DOI:** 10.1038/s41598-022-08131-1

**Published:** 2022-03-22

**Authors:** M. C. Cantor, David L. Renaud, Heather W. Neave, Joao H. C. Costa

**Affiliations:** 1 Dairy Science Program, Department of Animal and Food Sciences, University of Kentucky, Lexington, KY 40546 USA; 2 Department of Population Medicine, University of Guelph, Guelph, ON N1G 2W1 Canada; 3 Department of Animal Science, Aarhus University, 8830 Tjele, Denmark

**Keywords:** Zoology, Animal behaviour

## Abstract

Calves with Bovine Respiratory Disease (BRD) have different feeding behavior and activity levels prior to BRD diagnosis when compared to healthy calves, but it is unknown if calves who relapse from their initial BRD diagnosis are behaviorally different from calves who recover. Using precision technologies, we aimed to identify associations of feeding behavior and activity with recovery status in dairy calves (recovered or relapsed) over the 10 days after first antimicrobial treatment for BRD. Dairy calves were health scored daily for a BRD bout (using a standard respiratory scoring system and lung ultrasonography) and received antimicrobial therapy (enrofloxacin) on day 0 of initial BRD diagnosis; 10–14 days later, recovery status was scored as either recovered or relapsed (n = 19 each). Feeding behaviors and activity were monitored using automated feeders and pedometers. Over the 10 days post-treatment, recovered calves showed improvements in starter intake and were generally more active, while relapsed calves showed sickness behaviors, including depressed feed intake, and longer lying times. These results suggest there is a new potential for precision technology devices on farms in evaluating recovery status of dairy calves that are recently treated for BRD; there is opportunity to automatically identify relapsing calves before re-emergence of clinical disease.

## Introduction

Bovine Respiratory Disease (BRD), a disease of the upper or lower respiratory tract in cattle, is a welfare challenge to manage; it also is the leading cause for antimicrobial use on calf raising operations^1^. This disease complex causes inflammation of the respiratory tract^2^, coughing, pain, and a febrile response, resulting in the display of sickness behavior^3^. Some examples of sickness behavior related to BRD bouts in dairy calves are decreased feed intake recorded by automated feeders^4^, and reduced activity levels recorded by accelerometers^5^. Other sickness behaviors in calves, such as self-isolation behavior^6^, depressive behaviors with signs of physical pain^7^, labored breathing^8^, and lateral lying^9^ can be directly observed (e.g., by video recordings). Thus, it is likely that BRD bouts compromise a calf’s welfare and the time a calf experiences disease should be minimized^10^. Bovine Respiratory Disease Complex (BRD) also presents a sustainability issue since calves often require multiple antimicrobial interventions to treat the disease. For example, BRD calves often have poor response to additional antimicrobial interventions^11^, leading to chronic pneumonia^11,12^, and potentially death^11,12^. Specifically, in one study, nearly a fifth of initial BRD bouts treated were relapsed cases, and some of these calves died^11^; however this research was a non-controlled study and research is needed regarding the prevalence of relapsed BRD in dairy calves. Therefore, research is needed to identify if recovery status is associated with behavior in calves.

Sickness behavior in relapsed cattle is like that associated with the initial BRD diagnosis, where decreased feed intakes, signs of depression, lethargy, and labored breathing are observed prior to clinical re-presentation^13^. Indeed, precision livestock researchers determined that a reticulorumen bolus was more sensitive at detecting recovery status than using only clinical signs in beef cattle that recently received an antimicrobial intervention for BRD^14^. However, research is limited since precision technology devices were initially developed to alert for deviations in behavior before the initial diagnosis of clinical disease^15^. Calves that responded to the initial antimicrobial intervention should resume behavioral baseline behavior as convalescence approaches. In contrast, it is expected that calves failing to respond to antimicrobial treatment for BRD have depressed feeding behavior and reduced activity levels, but this research is still needed.

Sickness behaviors often occur prior to clinical presentation due to the motivational state activated by the immune system to initially combat an infection^3^. In dairy calves, the most sensitive indicator of BRD was lying time, when using a random forest algorithm and rolling averages in lying time as machine learning techniques^16^. Depressed feeding behavior and reduced activity levels were also observed a few days before diagnosis of metabolic diseases in lactating dairy cattle^17^. Similarly, decreased feed intake^18–20^, increased lying times^20^ and less lying bouts^21^ were associated with BRD status when compared to healthy calves. Furthermore, precision livestock researchers suggested that a combination of precision technology devices (automated feeder, accelerometer and a reticulorumen bolus) could detect BRD status in cattle several days earlier than staff^22^. Thus, we suggest that there is real potential to use precision technology devices to monitor for sickness behaviors to detect recovery status in calves. The use of sickness behavior to indicate recovery status in livestock may be a new frontier for precision technology devices that requires investigation.

The objective of this study was to evaluate if recovery status was associated with feeding behavior and activity levels in dairy calves for the 10 days after antimicrobial intervention. We also evaluated if recovery status was associated with relative changes in feeding behavior and activity (i.e., a calf increases or decreases its behavior relative to initial BRD diagnosis and antimicrobial therapy). We hypothesized that relapsed calves would show reduced feed intake, fewer steps, and lower acceleration activity index, while recovered calves would show greater relative changes in daily behavioral patterns in relation to the initial day of diagnosis, reflecting convalescence.

## Materials and methods

This study was conducted at the University of Kentucky Coldstream Research Dairy Farm in Lexington, KY from 28 May 2018 to 9 September 2019. This study was approved and deemed ethical for all experimental procedures by the University of Kentucky’s Institutional Animal Care and Use Committee approval number 2018: 2864. This study and manuscript were reported by ARRIVE guidelines and conducted following the ethics and quality standards of Strengthening the Reporting of Observational Studies in Epidemiology Veterinary Guidelines^23^.

### Management and feeding

Detailed information on calf management and feeding are outlined in supplementary material. Briefly, calves were trained to drink milk from an automated feeder (CF100, Forster Technik, Engen, Germany) at 3 days of age and were fitted with a leg-based accelerometer (IceQube, IceRobotics, Edinburgh, Scotland). Calves were housed in group pens of 6 ± 3 calves (mean ± SD) and offered up to 10 L (12.3% DM) milk replacer per day from the automated milk feeder (Cow’s Match Cold Front; Land O’ Lakes Animal Milk Products Co., Shoreview, MN) until 50 day of age. The automated feeder recorded each calf’s daily voluntary milk intake, average drinking speed, and all feeder visits. Feeder visits were either recorded as nutritive when a calf received milk (rewarded visits), or as non-nutritive visits when a calf was not eligible to receive milk (unrewarded visits)^4^. Calves were also offered calf starter (Special Calf Starter and Grower, Baghdad Feeds, Baghdad, KY) from an automated dispenser (Compact Smart, Förster-Technik, Engen, Germany), chopped alfalfa hay in a trough, and water from an automated waterer. For this study, references to feed intake refer to starter intake and milk intake collectively.

### Health exams

Calves were health scored daily at approximately 08:30 h from birth until 2 weeks post-weaning (87 ± 2.0 days of age) by 1 of 3 observers (inter-observer agreement κ > 0.90) for the following signs of health events: Bovine Respiratory Disease (BRD), diarrhea and sepsis as described in detail in Cantor et al., 2021^24^. Fleiss’ kappa was calculated every 4 months throughout the study by having all observers go to a commercial facility on the same day to score the health status of 40 calves where the health status of the calves was unknown to ensure unbiased agreement. Since the calves were followed daily for health events, the main observer was not blind to disease outcomes. However, to limit observational bias, the other 2 researchers were blind to antimicrobial interventions, and the farm staff administered all antimicrobial treatments to calves.

Signs of Bovine Respiratory Disease (BRD) were recorded by researchers using the Wisconsin Calf Health Scoring Chart.^25^ Researchers assigned scores (0 normal to 3 severely abnormal) on: abnormal presentation of cloudy nasal discharge and eye discharge, degree of ear tilt, degree of coughing, and degree of elevated rectal temperature in calves. Lung consolidation was recorded twice weekly for all calves using a portable linear rectal ultrasound (Ibex Pro, E.I. Medical, Loveland, CO) and 70% isopropyl alcohol as a transducing agent; lung lobes of each calf were evaluated by 1 of 2 observers (inter-observer agreement Cohens’ kappa; κ = 0.90). The ultrasound was set to a depth of 9 cm, frequency of 6.2 MHz, and gain of 23 dB (near 13 dB; far 36 dB).

We classified a BRD bout using two categories that were categorized as abnormal in the Wisconsin Calf Health Scoring Chart (score ≥ 5) and we required calves to have lung consolidation in any one lung lobe ≥ 3.0 cm^2^. A BRD bout diagnosis was represented on day 0 (“initial diagnosis”) as described in Buczinski et al., 2018^26^. All calves with a positive BRD bout received antimicrobial intervention on initial diagnosis; enrofloxacin was administered subcutaneously with dosage calculated by BW (Baytril, Bayer, Leverkusen, Germany; 1 ml/15 kg) according to the herd veterinarian protocol. From day 1 to day 9 after initial diagnosis, calves were health scored, but no clinical diagnosis was made (“post-treatment” period). From day 10 to day 14 after the initial diagnosis, all calves were assessed daily for recovery status from their initial BRD bout (“recovery classification period”). Calves who were abnormal on the Wisconsin Scoring system for at least three days during the recovery classification period, and had at least one lobe of lung consolidation at ≥ 3.0 cm^2^ during the recovery classification period were classified as failures (“relapsed”). Calves received a new antimicrobial intervention on day 15 as per the veterinary protocol for this research facility. Calves negative for the clinical signs described above during the recovery classification period were considered responsive (“recovered”). Relapsed calves were treated with tulathromycin on day 15 (Draxxin, Zoetis Animal Health, USA; 1 cc/45 kg, once at second diagnosis, subcutaneously), following the veterinary protocol of the research facility.

Body weights were recorded twice weekly using an electronic scale (Brecknell PS1000, Avery Weigh-Tronix LLC, and Fairmont, MN) from birth to 2 weeks post-weaning for all calves.

### Automated data recording

Daily summaries were automatically generated by the software for each calf every 15 min and transmitted to a data cloud wirelessly for the following behaviors: lying time, lying bouts, and total step count^27^. An acceleration activity index score was also generated daily by an algorithm of this accelerometer’s software (IceQube, Ice Robotics, Scotland). This algorithm evaluated each calf’s average daily rate of acceleration and daily step count to generate an activity index^28^.

The automated feeder’s software (KalbManagerWIN, Förster-Technik, Engen Germany) summed milk intake, average drinking speed, calf starter intake and milk feeder visits (rewarded and unrewarded visits) into daily summaries for each calf and transmitted the data to a data cloud associated with the automated feeder software.

The temperature and humidity were recorded every 15 min by a data logger (HOBO U23 Pro., Hobo, Onset Corp., Bourne, MA) placed in a pen in the calf barn and all data was automatically transmitted to a data cloud for the duration of the study (Supplemental Table S1).

#### Enrollment criteria

This observational case–control study was a subpopulation of an observational cohort study of 120 calves (73 heifers, 47 bulls). These calves were health scored daily by researchers for signs of Bovine Respiratory Disease (BRD), diarrhea and navel health from birth until 14 days after weaning, with the final health exam performed on calves at 87 ± 2.0 days of age (mean ± SD). All calves born at this facility were weighed within 12 h after birth with an electronic scale (Brecknell PS1000, Avery Weigh-Tronix LLC, Fairmont, MN). Calf birth weights were 39.42 ± 5.31 kg (mean ± SD). Only calves with successful transfer of passive immunity (> 8.0% BRIX) at 48 h of age, and those that were not a twin were enrolled. Any calf not meeting these requirements were excluded from the study.

All calves enrolled in this case–control study (38 of 120 calves) had an initial BRD bout diagnosis that was classified by researchers using the Wisconsin Calf Health Scoring Chart and lobar consolidation in any one lung lobe ≥ 3.0 cm^2^. All calves received an antimicrobial intervention (e.g., enrofloxacin) on the day of initial BRD diagnosis. All calves enrolled on this study (recovered and relapsed) had never had a clinical health event, or received antibiotics prior to BRD diagnosis on day 0. Initial diagnosis of BRD occurred between 3 ± 2 days of age (age of training to drink milk from an automated feeder) and 53 ± 2 days of age (age before weaning). We chose the preweaning phase since individual variation in feeding behavior during weaning might have introduced bias to our findings^29^.

#### Case–control classification and study population

Of the original cohort of 120 calves, 54 calves had BRD bouts and of these BRD bouts, we enrolled (n = 38) pair matched cases to controls; 19 recovered cases and 19 relapsed cases. The calves on this study had an initial BRD bout diagnosis at an average age of 32.0 ± 13.0 days (mean ± SD) and weighed 54.4 ± 9.7 kg. To limit bias, only clinical BRD signs (not behavior) were used to classify recovery status from day 10 to day 14 in these calves. Criteria for a case relapsed calf required the calf to have an abnormal BRD score for at least three days during the recovery classification period, positive clinical lobar pneumonia on ultrasound for the recovery classification period, and follow-up antimicrobial intervention (selection realized at random). Criteria for a control recovered calf required the clinical BRD score to be < 5 for at least 3 days during the recovery classification period, plus resolution of lobar pneumonia during the recovery classification period, complete technology data, and had to meet pair matching criteria. The pair matching criteria was same gender, age of the initial antimicrobial intervention no more than 2 days different, and birthdate within 6 weeks.

A power analysis identified that 17 relapsed calves were required to detect behavioral differences from recovered calves. Details on this calculation are provided in the supplementary methods.

### Statistical analysis

All statistical analyses were performed in SAS version 9.4 (SAS Institute Inc., Cary, NC, USA). Significance was reported at *P* ≤ 0.05. Descriptive analyses were performed, and data was verified for normality using the univariate procedure and probability distribution plots. Normality was also investigated by visually examining the residuals from the linear mixed models, and by testing covariance structures for model fit. One outlier relapsed calf was detected for abnormally low drinking speeds (greater than 3 SD from the mean) and out of the biological expected range; this pair was excluded from the drinking speed analyses.

Calf starter intake and unrewarded visits were not normally distributed and were transformed accordingly (calf starter intake: common log with correction factor of 10 g for calf starter intake; unrewarded visits: common log with a correction factor of 5 visits). The back-transformed geometric means minus the correction factors and the 95% CI are reported for both calf starter intake and unrewarded visits, with statistical significance reported on the modeled transformed values.

For all linear mixed models in this study, multivariable models were reduced by manual stepwise backward elimination, where predictors with a P-value < 0.30 were retained in the models. Season was evaluated in all models as a fixed effect and birthweight, Brix % serum IgG at 48 h of age, and calf age were tested as quantitative covariates. Due to the collinearity observed during univariate analysis for the covariates BRIX % IgG at 48 h of age and age enrolled on the feeder, these were evaluated for model inclusion separately. If BRIX % IgG at 48 h of age was not significant, age enrolled on the automated feeder was evaluated. Pair was a fixed effect in all models.

#### Effect of recovery status on average calf behavior

Linear mixed models were used to investigate the association of recovery status, and the recovery status x day interaction, with average feeding behavior including milk intake, drinking speed, rewarded visits, unrewarded visits, and starter intake, and activity of calves including total step count, lying time, lying bouts, and the acceleration activity index during the 10-day post-treatment period (day 1 to day 10; referred to in results as “post-treatment period”). A 10-day post-treatment period was selected based on lower Akaike’s criterion than all other lengths from 3-day to 9-day treatment periods. The recovery classification period was excluded from the post-treatment period to limit bias of finding behavioral differences during clinical presentation disease in relapsed calves. Models were designed with day as a repeated measure and calf as the subject with an autoregressive first order (AR1) covariance structure. Significance for recovery status x day interactions was explored, but since this was an exploratory analysis, no daily differences were reported.

#### Effect of recovery status on relative changes in calf behavior

Associations between recovery status and the recovery status × day interaction with relative changes in feeding behavior and relative changes in activity over the post-treatment period were tested with an identical modeling structure to that described above. We used PROC NPAR1WAY to confirm that averages and variances of feeding and activity behaviors were deemed similar on the day of initial diagnosis in all calves (e.g., recovery status was not yet evident). Since no differences were evident, day of initial diagnosis (day 0) was set as the behavioral baseline and deemed the 100% level for each behavior of each calf. Relative change was then calculated by dividing every calf’s individual behavior at each day (day 1, day 2, day 3, day 4, day 5, day 6, day 7, day 8, day 9 and day 10) by every calf’s behavioral baseline at initial diagnosis on day 0. Thus, relative changes reflect changes in daily behavioral patterns in relation to the day of initial diagnosis. Significance for recovery status x day interactions were explored, but since this was an exploratory analysis, no daily differences were reported.

## Results

### Study population

All recovered calves (19 of 19) resolved signs of BRD at 7.0 ± 3.0 (mean ± SD) days after the first intervention. Relapsed calves resolved signs of BRD (15 of 19) at an average 22.0 ± 5.0 days (mean ± SD) after the first antimicrobial intervention. Four relapsed calves did not resolve the initial BRD bout after the veterinary treatments were applied and were euthanized by veterinary recommendation. All euthanized calves were sent to a diagnostic lab (University of Kentucky Diagnostic Lab, Lexington, KY, USA); the preliminary cause of death was chronic pneumonia.

### Association of recovery status with behavior over the post-treatment period

**Figure 1:**
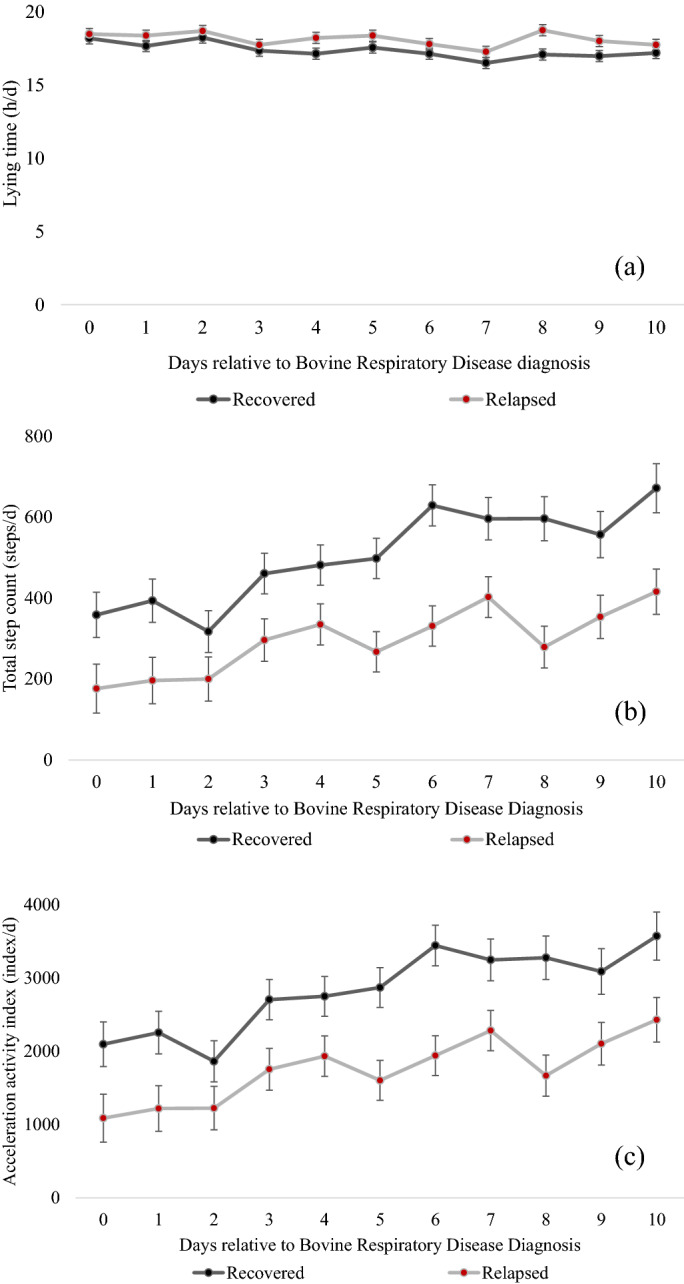
The association of recovery status^1^ with lying time (**a**), total step count (**b**) and acceleration index (**c**) (LSM ± SEM) for the day of diagnosis (day 0) and the 10 days after antimicrobial intervention for Bovine Respiratory Disease for preweaned, pair-matched calves (n = 19 pairs) offered 10 L of milk replacer/day by an automated feeder. ^1^Bovine respiratory disease (BRD) status was defined as a clinical score on Wisconsin Scoring system and lobar lung consolidation ≥ 3 cm^2^ and all calves were treated for BRD on day 0. Recovery status was defined as calves that either resolved symptoms of BRD within 10 days post-antimicrobial treatment (recovered) or had clinical BRD status from days 10–14 (relapsed). Significant differences by day are not reported.

The association of recovery status with average feeding behavior and activity levels during the post-treatment period are presented in Table 1, and significance of a recovery status × day interaction is also reported. Briefly, relapsed calves had less milk intake, slower drinking speeds, fewer unrewarded visits, less starter intake, and fewer lying bouts compared to recovered calves during the post-treatment period. There was a recovery status × day interaction for lying time (Fig. 1a), step counts (Fig. 1b) and activity index (Fig. 1c).

**Table 1:** Association of recovery status (recovered or relapsed) with feeding and activity behaviors of dairy calves (n = 19 matched pairs^a^) in the 10 days after initial Bovine Respiratory Disease diagnosis and antimicrobial therapy on d 0.

Variable	Recovered	Relapsed	SEM	*F-*value^e^	*P*-value (recovery status)^c^	*P*-value (recovery status * day)
Milk intake (L/day)	9.05	8.16	0.17	12.47_1,17_	**0.001**	0.41
Drinking speed (L/min)	1.02	0.81	0.05	6.72_1,15_	**0.02**	0.41
Rewarded visits (visits/day)	3.74	3.80	0.10	0.20_1,17_	0.66	0.52
Unrewarded visits (visits/day)	2.40 [95% CI 0.21–2.59]	1.53 [95% CI 0.35–1.55]	NA	6.07_1,17_	**0.02**	0.62
Starter intake (g/day)	137.50 [95% CI 11.25–263.74]	51.54 [95% CI 23.74–126.24]	NA	18.48_1,16_	**0.001**	0.63
Lying time (h/day)	17.37	18.14	0.15	11.33_1,17_	** < 0.001**	**0.02**
Lying bouts (bouts/day)	19.59	17.39	0.48	10.47_1,17_	**0.001**	0.59
Step count (steps/day)	505.43	295.80	21.78	40.85_1,17_	** < 0.001**	**0.01**
Acceleration activity index^b^	2834.49	1751.26	115.47	38.79_1,17_	** < 0.001**	**0.04**

Feeding behaviors (average drinking speed, milk intake, and number of rewarded visits) were recorded by an automated milk feeder from calves offered 10 L of milk replacer/day, and activity measures (lying time, step count, acceleration activity index^b^ and lying bouts) were recorded by a pedometer. Results are reported as least squares means^c^, or geometric means^d^ for unrewarded visits and starter intake.

^a^All calves were pair matched to healthy calves by age at diagnosis, birthdate, and gender. Bovine Respiratory Disease was defined as a clinical score of at least 5^26^ and lobar lung consolidation^27^.

^b^The acceleration activity index was generated by the commercial software algorithm (IceRobotics, Scotland) based on a calf’s daily average acceleration rate and step count.

^c^Significance are in bold *P* < 0.05.

^d^Non-normally distributed variables were transformed with log_10_ for significance *P* < 0.05.

^e^Superscripts refer to numerator and denominator degrees of freedom.

### Association of recovery status with relative changes in behavior over the post-treatment period

**Figure 2:**
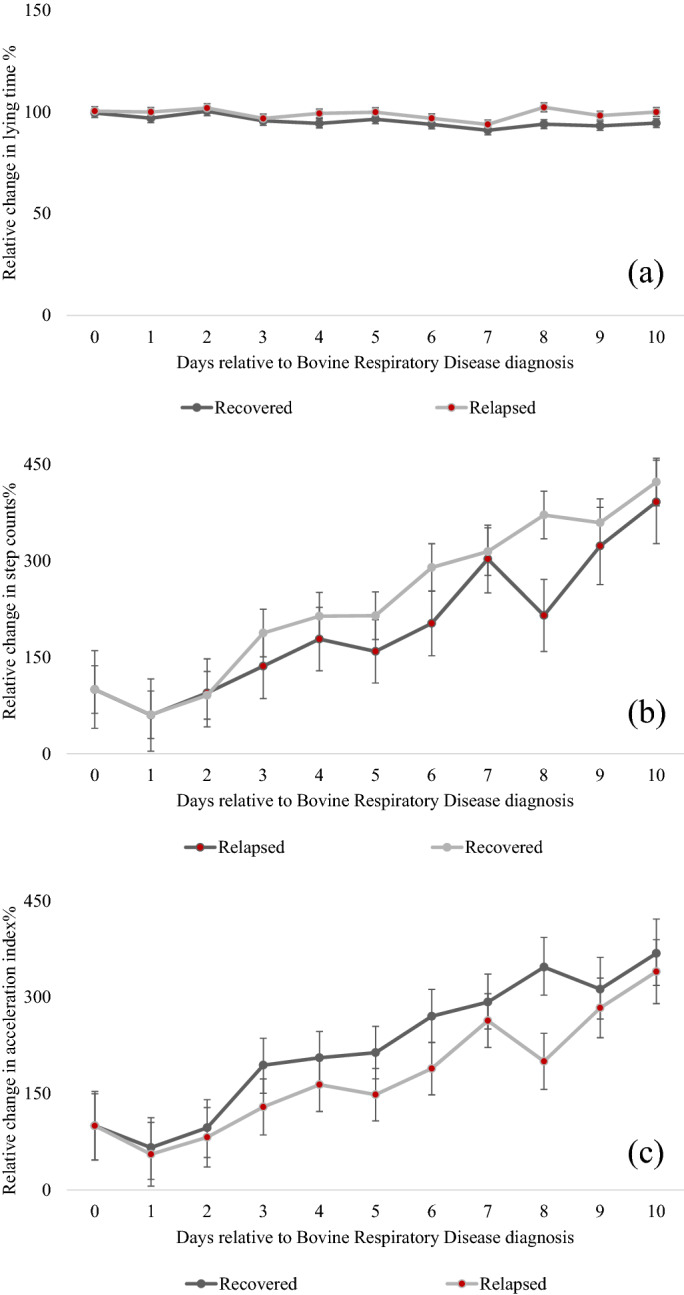
The association of recovery status^1^ with relative changes^2^ in lying time (**a**), total step count (**b**) and acceleration index (**c**) (LSM ± SEM) for the day of diagnosis (day 0) and the 10 days after antimicrobial intervention for Bovine Respiratory Disease for preweaned, pair-matched calves (n = 19 pairs) offered 10 L of milk replacer/day by an automated feeder. ^1^Bovine respiratory disease (BRD) status was defined as a clinical score of ≥ 5^26^ lobar lung consolidations ≥ 3 cm^2^ and all calves were treated for BRD on day 0. Recovery status was defined as calves that either resolved symptoms of BRD within 10 days post-antimicrobial treatment (recovered) or had clinical BRD status from days 10–14 (relapsed). ^2^Relative changes were calculated with day of antimicrobial treatment as a baseline (100%); relative changes were generated by dividing each day after BRD diagnosis (day 1 to day 10) by the baseline (day 0). Significant differences by day are not reported.

The associations of recovery status with relative changes in milk intake, starter intake, and activity behaviors of calves during the post-treatment period compared to day 0 are presented in Table 2 and significance for the recovery status × day interaction is also reported. There was a recovery status × day interaction for relative changes in lying time (Fig. 2a), relative changes in total step counts (Fig. 2b) and relative changes in acceleration activity index (Fig. 2c).

**Table 2:** Association of recovery status (recovered or relapsed) with relative changes in feeding and activity behaviors of dairy calves (n = 19 matched pairs^a^) in the 10 days after initial Bovine Respiratory Disease diagnosis and antimicrobial therapy on d 0.

Variable	Recovered	Relapsed	SEM	*F-*value^d^	*P*-value (recovery status)^c^	*P*-value (recovery status * day)
Relative change milk intake %	96.66	101.13	3.01	1.00_1,17_	0.33	0.91
Relative change drinking speed %	114.24	89.96	5.11	10.18_1,16_	**0.01**	0.22
Relative change rewarded visits %	105.58	110.07	4.24	0.50_1,16_	0.49	0.17
Relative change unrewarded visits %	105.31	103.27	1.95	0.54_1,18_	0.47	0.62
Relative change starter intake %	125.81	104.70	4.34	10.44_1,17_	**0.01**	0.32
Relative change lying time %	95.48	98.81	0.90	6.70_1,17_	**0.01**	**0.05**
Relative change lying bouts %	112.28	107.04	3.18	1.32_1,17_	0.27	0.20
Relative change step count %	229.81	185.96	27.61	1.24_1,18_	0.28	**0.01**
Relative change acceleration activity index^d^ %	216.36	168.11	22.38	2.28_1,18_	0.15	**0.01**

Relative changes referred to day 0 as a baseline. Feeding behaviors (average drinking speed, milk intake, and number of rewarded visits) were recorded by an automated milk feeder from calves offered 10 L of milk replacer/day, and activity measures (lying time, step count, acceleration activity index^b^ and lying bouts) were recorded by a pedometer. Results are reported as least squares means^c^.

^a^All calves were pair matched to healthy calves by age at diagnosis, birthdate, and gender. Bovine Respiratory Disease was defined as a clinical score of at least 5^26^ and lobar lung consolidation^27^.

^b^The acceleration activity index was generated by the commercial software algorithm (IceRobotics, Scotland) based on a calf’s daily average acceleration rate and step count.

^c^Significance are in bold *P* < 0.05.

^d^Superscripts refer to numerator and denominator degrees of freedom.

## Discussion

This study found an association between recovery status and behavior in dairy calves for the 10 days after antimicrobial intervention (post-treatment period). Calves that relapsed and required a second antimicrobial intervention had clear behavioral changes before the recovery classification period. There was an association between recovery status and milk intake, drinking speed, starter intake, unrewarded visits and lying bouts for the 10 days after antimicrobial intervention. Furthermore, there was an interaction of recovery status and day for lying time, step counts, and acceleration activity index, suggesting that relapsed calves were less active on some days compared to recovered calves. We also observed an association of recovery status with relative changes in drinking speed, calf starter intake, and significant interactions of recovery status and day for relative changes in lying time, step counts and index, suggesting the potential of these behaviors for future research regarding algorithm development to detect recovery status in calves. This research supports a new opportunity to passively monitor calves in recovery from BRD using precision technology devices.

Calves that recovered from BRD showed a response to the initial antimicrobial intervention much earlier than the time required for classification of recovery status. These recovered calves appeared to no longer experience the antigenic innate immune response associated with induced sickness behaviors^3^, including the reduced feed intake and evidence of lesser activity levels observed in relapsed calves in this study. Cytokine-induced sickness behavior is an antigenic response^3^ and a motivational state. The motivational state during a disease bout is an active response to infection^30^ to meet the metabolic demands of the febrile response^3^, increasing the chance of a calf’s recovery. In contrast, calves that relapsed from the initial antimicrobial intervention for BRD may have had the inflammatory pathway re-activated, leading to sickness behavior. Thus, a relapsed calf would lack motivation to seek feed by taking fewer steps and lying down for longer so that more energy resources are allocated to fighting an immune response. Moreover, the veterinary definition of recovery status following a disease response in livestock includes the absence of sickness behaviors such as depression, lethargy, and anorexia in conjunction with resolution of clinical disease^31^. Evidence from our study suggests that the absence of depressive states such as higher milk and starter intakes, faster drinking speeds, lower step count, and reduced lying time in more frequent lying bouts over a 10-day post-treatment period may indicate a calf in recovery from a BRD bout.

Recurrent BRD is a welfare challenge for the cattle industry and relapsed BRD calves require additional antimicrobial interventions^11,33^. Pre-weaning BRD is painful for the calf and is associated with depressed calf behavior^7^ as well as lowering the odds of survival past the first lactation^32^. We suspect a poor likelihood of full recovery following antimicrobial treatment for relapsed BRD bouts^11^ is due to the lag between recurrence of disease and re-emergence of clinical disease. This agrees with our findings, that relapsed calves had further depressed behaviors prior to the re-classification of disease compared to recovered calves. The welfare issue in recurrent BRD is its effects on the calf’s biological functioning, expression of natural behaviors, and a calf’s well-being, all components of good animal welfare and a life worth living^34^. Relapsed BRD also increases the likelihood of mortality^35^. Thus, we suggest that the use of milk intake, drinking speed, starter intake, lying time, lying bouts, step count, and acceleration activity index as a screening tool for assessing recovery status in calves, which may permit earlier antimicrobial interventions for calves that will relapse, improving calf welfare and overall health. Furthermore, differences in relative changes in calf starter intake and relative changes in drinking speed were associated with recovery status. Thus, there is the potential to use these behaviors for algorithm development to detect recovery status in calves in future research. We suggest that minimizing the duration of BRD status in calves is essential to promote calf well-being^10^.

Our relapsed calves had less milk intake, slower drinking speeds, fewer unrewarded milk feeder visits, less starter intake and lower activity levels including fewer lying bouts over the post-treatment period compared to recovered calves. A decline in feeding behavior and lower activity in general have been classified as classical sickness behaviors in lactating dairy cattle^19^. While the association of disease status with activity levels is less researched in calves^5^, it is well-documented that feeding behavior declines prior to disease diagnosis in calves^4,18,19^^,^^20^. Specifically, declines in feed intake and fewer unrewarded visits to the automated feeder were associated with sickness behaviors in dairy calves offered at least 10 L/day^4^. Our results are also in broad agreement with observations of relapsed beef cattle; depressed feed intake and longer lying times were indicative of poor response to antimicrobial treatments compared to recovered cattle^14^. One study challenged beef cattle with BRD associated pathogens and found that dry matter intake (i.e., feed intake) was the most robust feeding behavior to indicate recovery status compared to feeding rate and visits to the feeder (e.g., declined after disease and resolved earlier in cattle given an NSAID)^36^. This agrees with our findings, that of the feeding behaviors measured, relative changes in starter intake in calves could indicate recovery status. We suggest that a decline in calf starter intake is a sickness behavior in calves, as has been observed in dairy calves in BRD challenge studies^9^, and naturally occurring BRD in veal calves^37^ and beef cattle^38^. Future research should investigate the potential of using deviations in calf starter intake as an alert for recovery status in calves.

Despite an association of relative changes in calf starter intake with recovery status, we did not observe similar effects on relative changes in milk intake. This may be related to calves’ preference for milk over starter intake, especially when offered higher milk allotments (e.g., 10 L/day or greater)^29,39,40^. One study that weaned calves based on their voluntary starter intake found that multiple calves never reached the target grain consumption rate (e.g., 200 g/day) when they were offered high milk allotments^41^, suggesting that milk was a preferred feed source over stater intake. Alternatively, milk intake was lower over the post-treatment period in relapsed calves compared to recovered calves, suggesting that this variable could still be an indicator of recovery status, but perhaps day-0 is not the appropriate baseline to detect relative changes in milk intake.

Unrewarded visits were lower across the post-treatment period for relapsed calves compared to recovered calves. We suggest that the more active recovered calves were motivated to try to receive more milk than the 10 L/day offered compared to relapsed calves. Competition for access to the automated feeder is common in healthy calves, even when stocking densities are limited to smaller group sizes^42^. Moreover, unrewarded visits are an indicator of poor satiety in calves^43^. Thus, it is possible that relapsed calves had low motivation to access starter and used their limited energy resources to access milk.

In this study, we observed that relapsed calves had much lower activity patterns across the post-treatment period when compared to recovered calves. There is evidence that BRD is a painful disease for cattle^2,44^ and changes in activity levels in cattle may be partially due to the activated sickness response^3^. For example, a shift in lying behavior (including longer lying times and less lying bouts in mature dairy cattle) has been associated with painful diseases such as lameness^45,46^ and metabolic diseases in cattle^47^. Sickness behavior post-inoculation with BRD-associated pathogens in beef cattle has also reflected longer lying times and fewer lying bouts compared to controls^36^. For naturally occurring BRD, lower activity index scores in cattle were also observed^45^. Calves are known to become lethargic in the days prior to BRD diagnosis as characterized by longer lying times, fewer lying bouts, and declines in step activity^5^. For this study, we suggest that recovery status for BRD calves is indicated in multiple activity levels by day including fewer lying bouts across the 10-days, and reduced step counts and activity indexes on some days in relapsed calves. This was an exploratory study, so future research is necessary for algorithm development when using multiple behaviors to detect the most accurate deviations in behavioral patterns that could be indicators of recovery status in calves.

In this study we observed that feeding behaviors (measured by an automated feeder) and activity (measured by an accelerometer) can indicate recovery in calves for the 10 days post-treatment. One of the most common precision technology devices used for calf management is the automated calf feeder where more milk is offered without increasing management costs^48^, and programmable, individual weaning strategies have encouraged calf starter intake to promote rumen development^49^. Since sickness behavior indicates a motivational state, automated calf feeders and accelerometers may be useful for detecting BRD calves failing to respond to antimicrobial interventions. Similarly, accelerometers are a precision technology device used to detect cattle in estrus and are one of the most common precision technologies considered for adoption on a dairy farm^50^; this is primarily due to reduced labor costs compared with observing cattle for mounting behavior^51^. One of the greatest barriers for a producer to consider adopting a precision technology on farm is their familiarity with the technology and their perceived value of the data^52^. Thus, we suggest that producers may consider adoption of automated feeders and accelerometers to monitor calf health sooner than unfamiliar technologies.

In recent years, precision technology devices have been observed as an opportunity to also monitor the health of calves in a herd setting. Our study suggests that there is the potential to use these precision technology devices for a new frontier in animal health: to detect recovery status in BRD calves. However, one of the limitations of this study is that more work is needed to appropriately develop algorithms which combine behaviors to detect recovery status in calves. This was an exploratory study to demonstrate proof-of-concept, and we suggest that recovery status from an initial BRD intervention might be detectable when accounting for calf feeding behavior and activity levels. We caution that more research using machine learning techniques is needed before clinical recommendations can be made using these behaviors as indicators of recovery status in calves. This research is important to improve time to BRD re-intervention in calves.

To our knowledge, only one study has used precision technology devices as an indicator of BRD relapse status in cattle^16^. For beef cattle, the use of a reticulorumen bolus in real-time was successful at detecting an elevated temperature (e.g., febrile response) in cattle that relapsed from the initial BRD antimicrobial intervention^14^. Moving forward, researchers should incorporate precision technology devices into a collective working system to detect diseases. This is especially important since precision technology devices were designed to detect behavioral deviations at an individual level from a baseline, and current research has suggested that it is a combination of behaviors which results in the most accurate algorithms to detect calfhood disease^16^. For example, the initial onset of BRD was most accurately detected in bull calves when lying time, weight, and total visits to the automated feeder were included and recently lying time was validated as the most robust behavior for indicating BRD onset in calves^16^. The reason we investigated relative changes in behaviors in this study was to address if behavioral differences were robust enough to be significantly evident by individual behavioral changes in a calf. We suggest that there is a value to using behaviors collectively to indicate early onset of disease and recovery status in calves. Machine learning techniques using a precision technology device have previously been used to detect BRD in cattle^16,17,22,38^ and are a promising area for future research. Collectively, we suggest that there is potential to detect recovery status of dairy calves using sickness behaviors, where relative changes in drinking speed, relative changes in calf starter intake, and relative changes in lying time might be especially useful behaviors. Future research should quantify the capability of multiple behaviors recorded by precision technology devices to detect recovery status in calves in real-time.

## Conclusions

The results of our study suggest that recovery status in calves was associated with feeding behavior and activity levels recorded by precision technology devices. Recovered calves showed signs of improved behavioral responses after their initial antimicrobial intervention for BRD; these calves showed greater feed intakes over the 10-day post-treatment period and were more active on some days when compared to relapsed calves. In contrast, relapsed calves expressed sickness behavior over the post-treatment period, including less milk and starter intake, slower drinking speeds, fewer unrewarded visits and lying bouts, and longer lying times. Relative changes in drinking speed and calf starter intake might be useful for future algorithm development in the detection of relapsed BRD calves. Behaviors in relapsed calves that were lower during the post-treatment period (such as milk intake, unrewarded visits, and lying bouts) may also be useful but different behavioral baselines should be explored. Future research is required to determine which combination of behaviors, and which behavioral baseline, are the most clinically actionable to develop an algorithm which accurately alerts for recovery status in calves.

## Supplementary Information

Supplementary Information.

## Data Availability

Data collected and analyzed with SAS code are available electronically in the Mendeley data repository. 10.17632/d7bz9bhxv5.1.
