# Supplementary material for: Feeding behavior and activity levels are associated with recovery status in dairy calves treated with antimicrobials for Bovine Respiratory Disease
Source: Sci Rep. 2022 Mar 22;12:4854. doi: 10.1038/s41598-022-08131-1 (PMC8940924; doi:10.1038/s41598-022-08131-1)
Supplement: Supplementary file 1 — Supplementary Information. [file 41598_2022_8131_MOESM1_ESM.docx]

**Supplemental Methods**

### Calf management procedures

All calves received colostrum within 6 hours after birth and were placed in an individual pen until a strong suckle reflex was presented. Calves were fed 6 L/d of milk replacer (Cow’s Match Cold Front; Land O’ Lakes Animal Milk Products Co., Shoreview, MN) by bottle prior to feeder enrollment. Calves were fitted with an RFID tag in the left ear for identification by the automated feeder, and a pedometer (IceQube, IceRobotics, Edinburgh, Scotland) was attached above the metatarsal of the rear left leg using a Velcro band to track activity behaviors. Calves were trained to suckle milk from the automated feeder (CF100, Forster Technik, Engen, Germany) at an average age of 3.0 ± 1.5 d. Calves were assisted at the automated feeder every 12 h until they visited the automated feeder independently in a maximum of 4 assisted feedings.. The automated milk feeder was in a group pen (4.57 × 10.67 m) and the stocking density was 6 ± 3 calves (mean ± SD); calves were moved to an identical adjacent pen with the same stocking density using dynamic flow at an average age of 45.0 ± 3.0 d (mean ± SD) before weaning.

Calves were allotted up to 10 L (140 g/L) milk replacer/d from the automated milk feeder (Cow’s Match Cold Front; Land O’ Lakes Animal Milk Products Co., Shoreview, MN) for 50 d, reduced to 50% allotment for 14 d, and then reduced to 20% allotment for an additional 7 d until complete weaning at 70 d. A separate automated calf starter feeder (Compact Smart, Förster-Technik, Engen, Germany) was present in each pen and contained calf starter (Special Calf Starter and Grower, Baghdad Feeds, Baghdad, KY); calves were also offered chopped alfalfa hay in a trough (1.83 × 0.33 × 0.16 m) and water with an automated waterer. Both the automated milk feeder and the calf starter feeder were calibrated weekly according to manufacturer instructions.

### Sample size calculation

For the power analysis, we assessed the literature for the most conservative relapse-to-antimicrobial-intervention-rate when treating non-specified BRD bouts in calves as an estimate of the incidence rate of BRD relapse in the dairy calf population. The most conservative literature was Welling et al. (2020)^11^ who observed 16% (19 of 117) of BRD calves relapsed from their first antimicrobial intervention for Bovine Respiratory Disease. To calculate the incidence of relapse-to-antimicrobial-intervention-rate for the herd in this study, 2 researchers (Cohen’s kappa= 0.95) scored calves twice weekly for BRD (32 of 80 BRD calves) for the 9 months prior to this study; farm staff administered antimicrobial interventions based on veterinary protocol and clinical signs of BRD^25,26^. The relapse-to-antimicrobial-intervention-rate was 43.75% (14 of 32). Therefore, at 80% power, with half-widths of 0.05, we required 17 relapsed calves to detect behavioral differences from recovered calves.

**Supplemental Table S1**. The average (mean ± SD) seasonal temperature and humidity of the preweaned calf barn for a cohort of 120 calves health scored daily for Bovine Respiratory Disease Complex

| Time of Year | Temperature | Humidity |
| --- | --- | --- |
| Winter  December to February | 8.44 ± 5.07 °C | 76.52 ± 9.25 % |
| Spring  March to May | 13.13 ± 7.99 °C | 68.04 ± 12.11 % |
| Summer  June to August | 24.93 ± 3.74 °C | 75.38 ± 14.08 % |
| Autumn  September to November | 14.77 ± 9.20 °C | 81.51 ± 13.65 % |
